# EBRAINS Live Papers - Interactive Resource Sheets for Computational Studies in Neuroscience

**DOI:** 10.1007/s12021-022-09598-z

**Published:** 2022-08-20

**Authors:** Shailesh Appukuttan, Luca L. Bologna, Felix Schürmann, Michele Migliore, Andrew P. Davison

**Affiliations:** 1 Université Paris-Saclay, CNRS, Institut des Neurosciences Paris-Saclay, Saclay, 91400 France; 2 Institute of Biophysics, National Research Council, Palermo, 90143 Italy; 3 Blue Brain Project, École polytechnique fédérale de Lausanne (EPFL), Campus Biotech, Geneva, 1202 Switzerland

**Keywords:** Computational neuroscience, Data retrieval, Data provenance, Documentation, Experimental data

## Abstract

We present here an online platform for sharing resources underlying publications in neuroscience. It enables authors to easily upload and distribute digital resources, such as data, code, and notebooks, in a structured and systematic way. Interactivity is a prominent feature of the Live Papers, with features to download, visualise or simulate data, models and results presented in the corresponding publications. The resources are hosted on reliable data storage servers to ensure long term availability and easy accessibility. All data are managed via the EBRAINS Knowledge Graph, thereby helping maintain data provenance, and enabling tight integration with tools and services offered under the EBRAINS ecosystem.

## Introduction

A major shortcoming in computational approaches to neuroscience, such as modelling/simulation and data analysis, is the absence of a widely used and well-defined system for sharing code and data that would enable researchers to easily access the data resources employed in published studies and understand in detail the provenance of published results and figures. This impedes community-based, collaborative modelling efforts, reduces the utility of published models within the neuroscience community, and hinders the reproducibility of data analyses.

Existing tools and services, such as ModelDB (Hines et al. 2004), Open Source Brain (OSB) (Gleeson et al. 2012), and NeuroMorpho.org (Ascoli et al. 2007), make models, code and/or data available to the scientific community, with each attempting to fulfil a specific need. For example, ModelDB is a repository of published models with code that allows users to replicate at least a single figure from the corresponding publication. It is not intended to tackle issues regarding access to experimental data, and therefore does not, in general, hold information about data used to tune, validate or simulate the model. Similarly, OSB is a model repository that focuses on model formats and simulator-independent model representations, but not on prioritising the data underlying the models. NeuroMorpho.org is a curated inventory of digitally reconstructed neurons associated with publications, but doesn’t link with other data such as electrophysiological recordings or models of the reconstructed neurons. More general services such as Figshare (Hahnel 2013), Open Science Framework (Foster and Deardorff 2017), or (Dillen et al. 2019) have much less structured records, with limited domain-specific metadata. Finally, applications/infrastructures specifically addressing reproducibility and transparency in scientific publishing (e.g., eLife RDS, Galaxy, Repro Zip) require possibly demanding *ad hoc* installations, configurations and maintenance operations (Konkol et al. 2020).

EBRAINS Live Papers are interactive documents bringing together code, models, and data. They can be stand-alone publications, but in general complement published scientific articles. Interactivity is a prominent feature of the “Live Papers” with several integrated tools and services that allow users to download, visualise or simulate data, models and results presented in the corresponding publications. By bringing together the various resources underlying computational approaches in neuroscience publications (whether modelling/simulation or data analysis), they provide a more complete picture of the original researchers’ workspace. By virtue of being developed within the EBRAINS infrastructure, Live Papers take advantage of EBRAINS platforms and services for data and model integration and are able to effectively leverage the Knowledge Graph (KG) database for storing all information. The KG, being a graph-based database, interlinks all data units thereby readily offering a high degree of data provenance.

Below we describe the various features and functionalities currently made available through the Live Papers platform, and also how authors can distribute their own resources by creating and publishing live papers. Our goal is to demonstrate to the scientific community the utility of building live papers to complement their publications, and to develop them not *post facto*, but rather as a tool to support their manuscript submissions by making available information that could assist reviewers in making a more informed evaluation (Bailey et al. 2016). Computational studies in neuroscience, being potentially fully deterministic, lacking the intrinsic variability of experimental studies, should be easily reproducible. In reality, this is often not the case, which to a large extent is due to the black box situation arising from missing or incomplete access to data and documentation. With the concept of Live Papers presented here, we hope to take significant strides towards resolving these problems.

## Overview

In this section we begin with a brief overview of the EBRAINS infrastructure, and move on to describing the various features and functionalities that are currently available for live papers.

### EBRAINS Infrastructure

An online platform for sharing digital resources and publishing interactive, scholarly documents places several requirements on the underlying infrastructure. Resources and documents must be persistent—reliably available over a long time period. Tools for visualising or re-using resources (models, code, data) should be available, reliable, give reproducible results, and be underpinned by adequate computational resources. Accurate and complete metadata must be available.

EBRAINS is a new digital research infrastructure developed under the Human Brain Project and now on the European Strategy Forum on Research Infrastructures (ESFRI) roadmap. It aims to bring together an extensive range of data, alongside tools to help analyse and utilise these data. Among the key tools and services provided by EBRAINS are (i) long-term, archival-quality data storage, provided through an agreement with the Swiss National Supercomputing Centre; (ii) a state-of-the-art metadata store, the Knowledge Graph; (iii) a wide range of tools for modelling, simulation, and data analysis at different scales and levels of abstraction; (iv) facilitated access to high-performance computing systems; (v) collaborative workspaces (the Collaboratory) with fine-grained access control for the documents, code and data for a given project. It is this infrastructure, fulfilling the requirements outlined above, that makes the Live Paper platform possible.

The Human Brain Project will be completed in 2023, but the tools and services developed under it will continue to exist as part of EBRAINS. It will continue to be funded by the participating countries, with national nodes being set up in multiple European countries, and with further funding from the EC until at least 2026, to ensure continued availability of these services. Live Papers is one of several services in the EBRAINS ecosystem, and their sustainability is the primary objective of EBRAINS AISBL.

### Live Paper Resources

The live papers allow for diverse types of resources to be presented, with practically no limitations. For computational modelling studies, we have found that the most common resources being distributed comprise the following:

- Electrophysiological Recordings

**Fig. 1:**
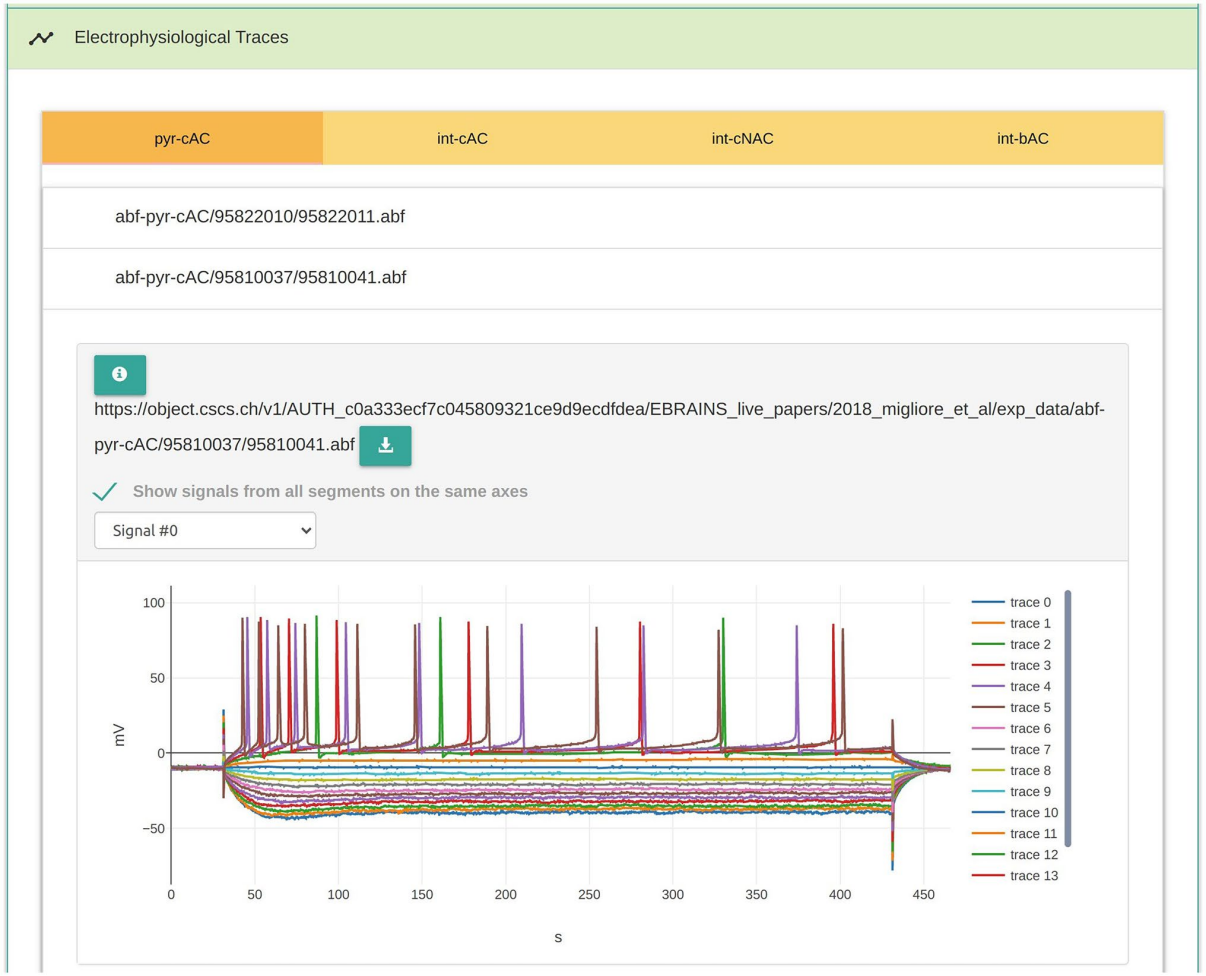
The electrophysiology resource section embeds an interactive visualiser for each electrophysiology data file. This enables users to visualise and to interact with the data live from within the document

Data obtained from experimental recordings often lay the foundations for modeling studies. These recordings can be obtained from a variety of different experimental techniques and protocols, such as intracellular recordings via sharp microelectrodes or patch clamp setup, calcium or dye imaging studies, and so on. The Live Paper platform provides for listing and access of all kinds of experimental data. A typical characteristic of experimental data is that they are often recorded as time series. The platform integrates an online visualiser for such data, as seen in Fig. 1, without requiring users to download them. It handles all data formats supported by the Neo library (Garcia et al. 2014). The Live Paper platform currently integrates with the EBRAINS Knowledge Graph and the Allen Brain Atlas (Jones et al. 2009) to enable authors to link to previously registered electrophysiological recordings from these databases.

- Neuronal Morphologies

Computational studies involving modeling of single neurons or networks of neurons typically employ digitally reconstructed neuronal morphologies for configuring the anatomical structure of model neurons. In the case of neuronal circuits and networks, a multitude of such neuronal morphologies might be employed. As cellular biophysics is greatly affected by the anatomical architecture, it is essential to have access to such data to ensure reproducibility of results. The live paper tool allows authors to list all the morphologies associated with their study, and allow interested users to download these. Authors may also link to established repositories such as NeuroMorpho.Org and the Allen Brain Atlas. The platform also integrates a 3D morphology viewer that users can use to explore and examine the morphologies (Bakker and Tiesinga 2016).

- Model Source Code

One of the fundamental requirements to ensure reproducibility of a computational study is access to the original source code used by the authors to arrive at the reported results. It is almost always non-trivial to reconstruct a model from scratch solely based on the model description provided in publications. Without access to the model source code, all inferences and conclusions have to be evaluated at face value without any scientific appraisal. This has encouraged most scientific publishers to ensure that the source code underlying modeling studies be made publicly accessible, or at least available on request.

**Fig. 2:**
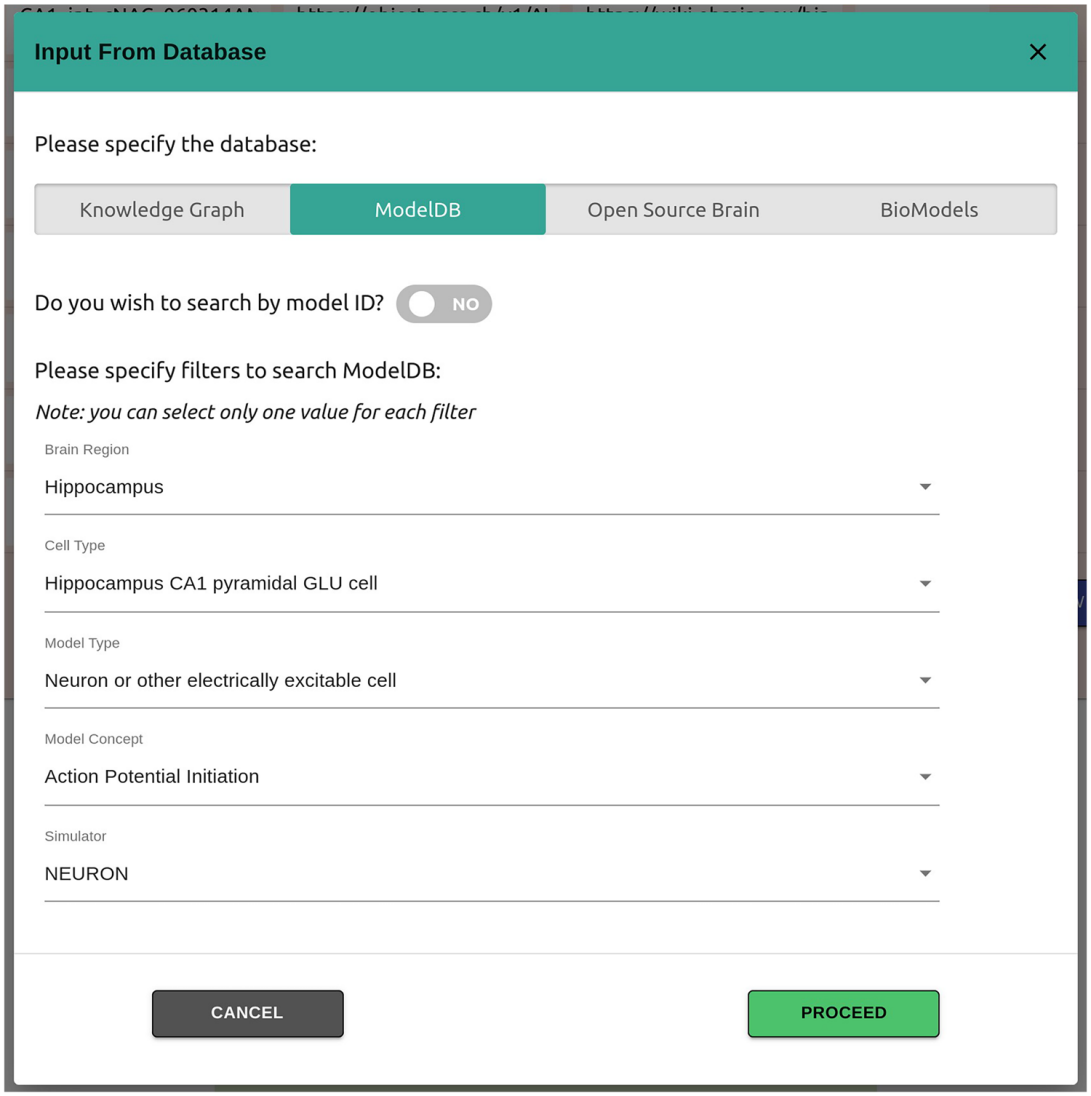
Widgets allow live paper creators to import resources from established neuroscience repositories. The figure illustrates how models can be imported from the EBRAINS Knowledge Graph, ModelDB, OSB or BioModels. Filters are available for shortlisting models based on specified criteria, or alternatively selected based on their identifiers

**Fig. 3:**
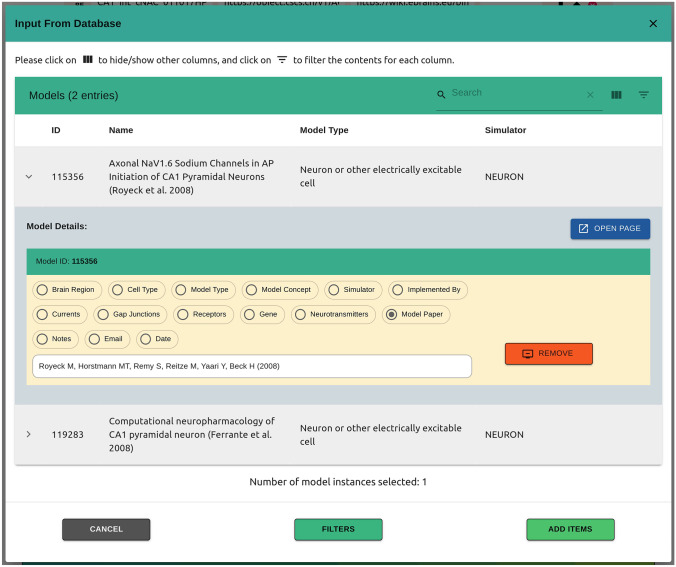
Based on the specified filters, data from the specified neuroscience repository, here ModelDB, are retrieved and tabulated. Authors are provided with relevant metadata for these entries, and are able to add these items to the live paper

Live papers allow authors to distribute the source code for their models. As shown in Figs. 2 and 3, they also enable importing and linking to corresponding entries in other neuroscience repositories such as ModelDB, OSB and BioModels, in addition to the EBRAINS Knowledge Graph. Additionally, live papers are closely integrated with the EBRAINS Model Catalog and KG. This permits authors to link their models to corresponding entries in the model catalog, which then provides additional information on the models, including outcomes of any validation tests they might have undertaken using the EBRAINS Validation Framework.

- Jupyter Notebooks

Jupyter Notebook is a free, open-source interactive web application that supports several programming languages. Over the past few years, notebooks have become the foremost choice for researchers to demonstrate their computational work. It allows them to aggregate software code, simulated outputs, documentation and other relevant resources into a single document Perkel (2018). A highlight of Jupyter notebooks is their ability to capture output, and thereby act as the equivalent of laboratory notebooks for computational researchers. A Jupyter notebook basically creates a snapshot of the actions of the original authors and the outcomes they witnessed, and in principle should enable any other user to follow the same footsteps to arrive at the same results. Well designed Jupyter notebooks can also serve as documentation of a study for the better understanding of reviewers and readers. The Live Paper platform allows authors to add Jupyter Notebooks, and enables users to launch these within the EBRAINS Collaboratory by simply clicking on each of them. This allows users to readily explore and execute the contents of these notebooks, without requiring any further setup, and can help in reproducing simulations and analysis from the original study.

Live papers are highly flexible and can easily accommodate other types of resources, not covered above. Authors can create listings of any kind of data, thereby enabling access to the scientific community for their perusal and further reuse in scientific studies.

### Live Paper Builder Tool

**Fig. 4:**
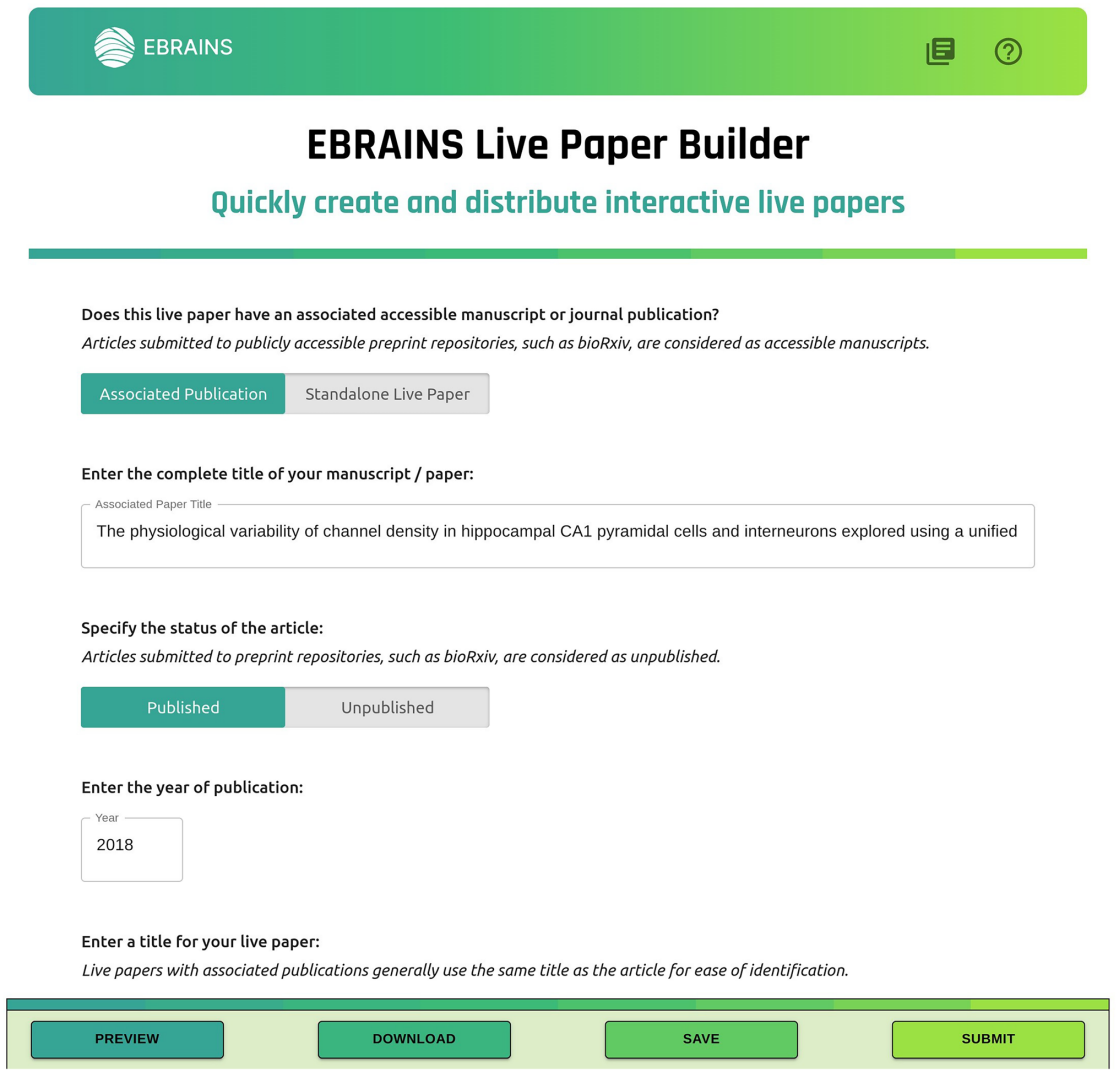
The browser-based live paper builder tool allows users to fill information in a form-based interface to create live papers. This process does not require any programming or web development skills and is very intuitive. Live papers with any level of complexity can be developed from within this tool

Initially, the process of creating live papers involved the authors having to download an HTML page template and editing this as required to incorporate all the data resources. This was somewhat arduous and required some knowledge of web development. Such an approach was evidently a barrier to wider adoption of the live paper concept, and so we have now developed a live paper builder tool that allows authors to construct live papers by simply entering information into a form based interface. Fig. 4 shows a snapshot of the user interface for entering the required information.

Where the live paper is associated with a traditional publication or preprint, metadata about the associated publication can be automatically extracted from the PDF version or entered manually. The tool provides widgets for furnishing information on the various kinds of resources discussed earlier. Additionally, it also provides more advanced users the ability to add custom functionality by specifying custom HTML or Markdown content. The latter ensures that the live paper builder tool does not limit the complexity of live papers that can be developed using it.

As seen in the lower toolbar in Fig. 4, the tool allows authors to preview changes, to download the resultant HTML file as well as the project file, with all data saved in JSON format, and to save the project to the KG. This allows users to develop the live papers across multiple sessions and/or enhance them over time, by either loading the previously downloaded project files, or via selecting from a list of live paper projects from the KG that the user has access to. Once the live paper development is completed, users can raise requests, from within the tool, for them to be published on the platform.

### Provenance Tracking

The EBRAINS KG is a multi-modal metadata store that aggregates information from various fields of brain research into a single interconnected network of data. It integrates experimental data, models, software and other related resources into a graph based database, and links these data units by their relationships to each other. This enables identifying all relevant resources associated with a particular research object. For example, for a given electrophysiological recording stored in the KG, it would be possible to retrieve all metadata associated with that recording, such as information on species, cell type, data modality, and additionally other linked research objects such as experimental protocols, software, and models that have employed the given recording in their development or validation. The KG also allows for connecting datasets to relevant software tools that can help with analysing and visualising the contained data.

The Live Paper platform stores all the information provided within the live papers in the KG. While creating the live paper, users have the option of selecting existing KG resources (e.g. models, experimental recordings) or, alternatively, specifying new resource units not currently available in the KG. We encourage the former approach, and accordingly guide users to tools/processes for registering resources on the KG, such as the EBRAINS Model Catalog for registering new models and the EBRAINS Data Curation Service.

### Limiting Visibility: Password-protection

It is understandable that authors might prefer to have their resources kept private prior to publication. At the same time, the resources made available in the live paper are often valuable to potential reviewers for a better assessment of the manuscript under consideration. To allow authors to develop and share live papers with reviewers, without risking reviewer anonymity by requiring reviewers to create an account, live papers offer the possibility of restricting their accessibility via password-protection. Authors can share the live paper URL and the associated password with journal editors, who can then pass them on to reviewers. This could also help address the data availability requirements posed by journal publishers at the time of manuscript submissions. Password-protected live papers are not listed on the Live Paper platform, and can only be accessed via their direct URLs and assigned password. To further ensure preservation of reviewer anonymity, the web server logs for the platform do not store any information allowing the identification of individual users, and are anyway not accessible to live paper authors.

### Issuing DOIs

The EBRAINS platform issues Digital Object Identifiers (DOIs) for curated research objects that are published through its Data and Knowledge Services, including experimental datasets and Live Papers. Before publication, live papers go through a quality control process; described in the following section. Issuance of a DOI further assists the citation of published data and models, thereby incentivising authors to publish and share such resources, and ensuring that they are duly acknowledged when these resources are reused.

## Live Paper Life Cycle

Below we outline different phases of the live paper life cycle, from development to publication and usage.

### For Authors: Development to Publication Phase

A live paper can be developed as a stand-alone resource, or as a supplement to an existing or future publication. In this second case, the requirement is for the availability of an associated manuscript. This may be an already published manuscript, in which case authors could point to the article on the journal’s website, or prior to publication hosted on a preprint server such as bioRxiv (Sever et al. 2019). The live paper itself can be created by any of the original authors on the study, or by a third person, in which case the publication of the live paper will require an approval from one of the original authors.

**Fig. 5:**
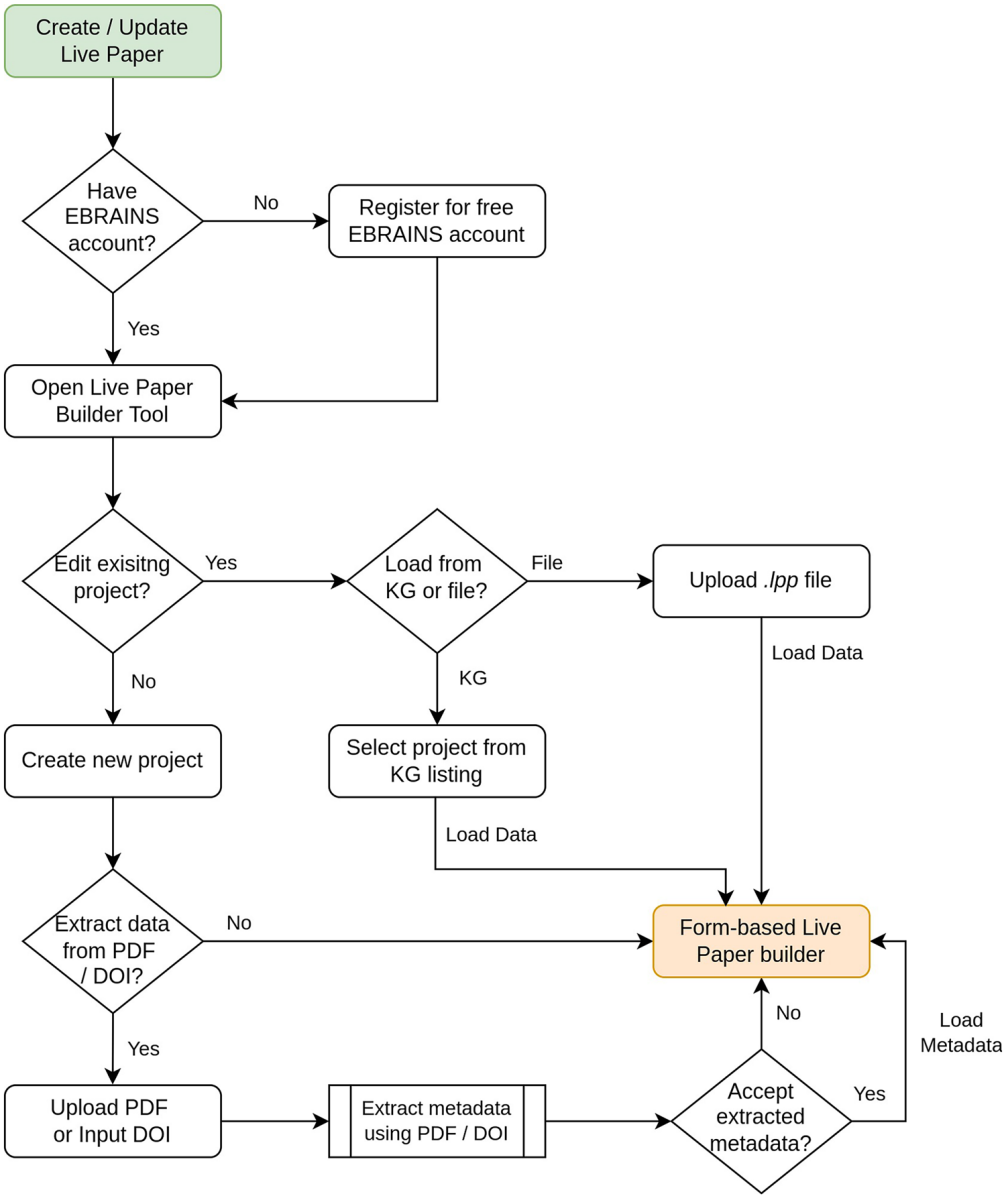
Flowchart depicting the steps involved in creating a live paper. Live papers can either be initiated from scratch, or have the publication related metadata extracted and auto-populated from the associated publication. Live papers are often developed and updated over multiple sessions, and can therefore be saved to KG at any time, or also downloaded locally

Figure 5 presents the steps involved in the live paper creation process in the form of a flowchart. The first step towards creating a live paper is to apply for an EBRAINS account. Interested users with a current affiliation to an academic institution can directly create an account at: https://ebrains.eu/register/. Users without such an affiliation may request an account. Once they have an EBRAINS account, they can access the live paper builder tool at: https://ebrains.eu/service/live-papers/. On opening the tool, users are given an option to either begin creation of a new live paper, or to continue working on an existing project - by loading a previously downloaded project file or selecting a live paper project saved in the KG.

When starting a new live paper project, users are given the option to upload the PDF file of an associated manuscript. The tool then attempts to auto-extract all the necessary publication related information from the uploaded file. We employ GROBID (Lopez et al. 2009), a tool for extracting metadata from scholarly publications, for this purpose. As another option, users can specify the DOI of the published article, and the tool will retrieve the associated metadata. Note that this second method is typically more accurate, but can retrieve limited info using the DOI. Alternatively, authors can manually enter all this information pertaining to the publication.

**Fig. 6:**
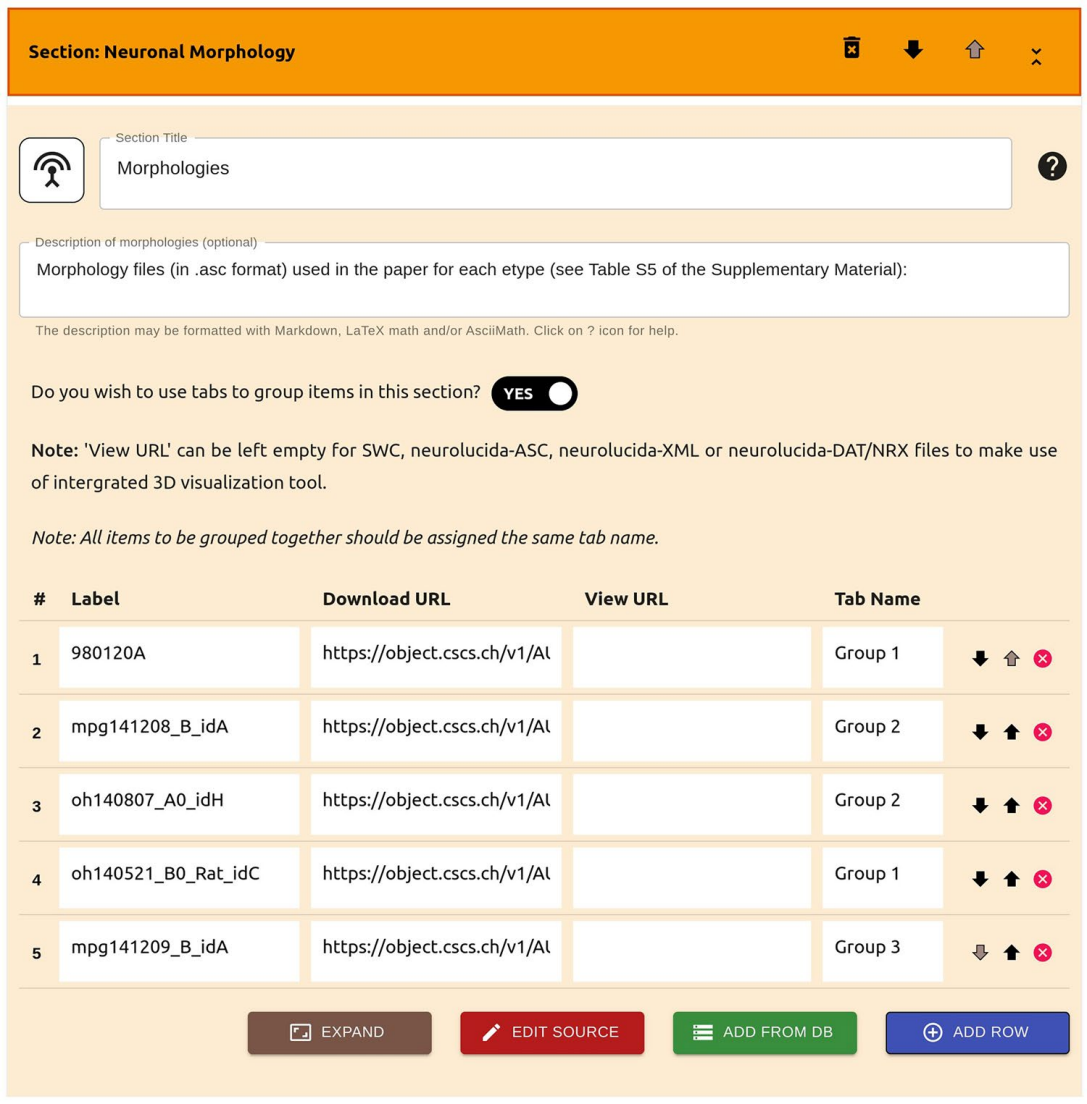
A widget for allowing users to input a collection of neuronal morphologies. The data can be input either by manually filling the fields, or by directly updating the underlying JSON content. Each widget additionally allows users to select an icon, specify a title and description, and offers the option of grouping items into categories. Widgets also enable the import of data from well-known neuroscience repositories

Widgets are provided for listing the different types of resources discussed earlier. Each widget requests, for each item in the listing, information such as the download URL for the resource, the label to be used in the listing, and optionally other resource-type specific information, such as the URL to a Model Catalog entry for model source code resources. An example of such a widget for listing morphologies is illustrated in Fig. 6. The widgets generally offer multiple input formats, with an eye to support both manual entry of information, as well as to assist copying over common programming constructs, such as lists and dictionaries. We have recently incorporated a spreadsheet-based input tool that makes it simpler to enter multiple entries at once via the GUI. The user does not need to carry out any additional tasks to integrate the visualisation tools such as the morphology viewer and neural activity visualiser. Authors are also requested to select a licensing policy to apply to the resources that they have listed on the live paper.

Live papers, during their development phase, are not publicly accessible. They have their access restricted to users belonging to a group, as defined by membership of a user-selected workspace (known as a ‘Collab’) in the EBRAINS Collaboratory. The authors, at the time of saving the live paper in the EBRAINS KG, therefore need to specify a Collab for the live paper being developed; this could be one of their existing Collabs or they can create a new one. Members of the Collab with administrator permissions can add or remove team members, and can therefore control visibility of the under-development live paper. During the development phase, all the data resources are controlled by the authors, including their storage locations. The live papers can continuously be updated in this phase.

**Fig. 7:**
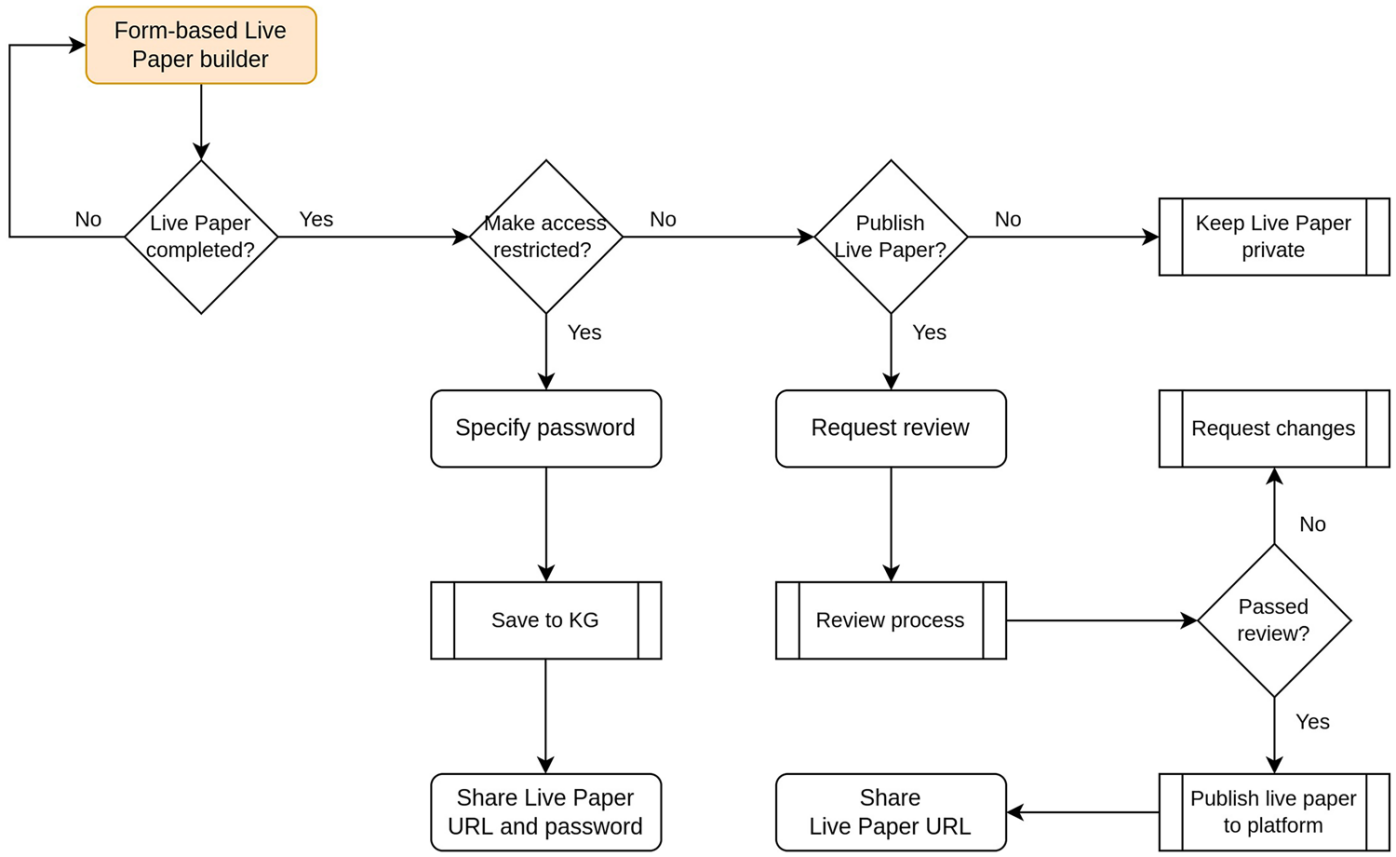
Flowchart depicting the workflow for processing a live paper once all the contents have been finalised. Authors can request to make the live paper password-protected, whereby access is restricted, or to have it published publicly

Once completed, the authors can submit the live paper for publication, after which it will undergo a curation process for the purposes of quality control. Understandably, it is not feasible to verify that a given live paper contains every single resource employed in an associated publication. This aspect is potentially best assisted by reviewers during the peer-review process of that publication, and we discuss this further later. It should be noted that live papers are not peer-reviewed from a scientific perspective. For now, the live paper curation process primarily involves verifying that all contained resources are actually accessible and that these are hosted on reliable data storage repositories. Resources hosted on authors’ or universities’ own websites are copied to the EBRAINS archival data repository, to ensure long term accessibility and availability of these resources. The URLs within the live papers are automatically updated to reflect these new storage locations. Resources hosted on other established neuroscience data repositories, such as ModelDB, Open Source Brain, BioModels, NeuroMorpho.org and the Allen Brain Atlas, are not duplicated, but instead we link directly to the corresponding entries in these repositories. Fig. 7 illustrates, through a flowchart, the steps involved in publishing a live paper.

Once the live paper metadata and contents have been verified, we publish the live papers on the platform. This makes it available to everyone. Once published, the process to make further changes to the live papers by the authors will require to again undergo the curation process to verify any changes. Hence it is strongly encouraged to request publication only once the contents are finalised. It should be noted that password-protected live papers, described earlier, are not considered as “published” and therefore do not undergo the curation process until submitted for publication.

### For Users: Post-publication Phase

Published live papers are categorized by year and listed on the Live Paper platform. All published live papers can be accessed freely by the scientific community. These can be freely accessed without an EBRAINS account, although certain integrated tools for re-running analyses or simulations might only be available for registered users owing to the need for accessing EBRAINS computing resources. For example: certain resources, such as Jupyter notebooks, can be easily copied over to the user’s own workspace in the EBRAINS Collaboratory, where they can effect changes and afford further explorations. Analysis and simulations can therefore be run on the cloud via the EBRAINS infrastructure. This feature requires the user to have an EBRAINS account to avail of computational resources. As mentioned previously, it is quite simple to request for an EBRAINS account, and would provide the user access to several other tools and services as well.

Users are free to access and use the resources provided in the live papers under the license terms specified by the authors of the live paper. Any additional queries or requests regarding the provided resources should be communicated to the live paper authors. For any issues regarding usability or accessibility of resources, users are requested to contact https://ebrains.eu/support for further assistance.

## Discussion

Live Papers are intended at making neuroscience publications more valuable to the scientific community by offering a holistic view of the various digital components of a publication, such as data analysis code, the data underlying model development, or simulation results. The availability and accessibility of underlying code and data will enable reviewers and other scientists to better evaluate a given model or analysis. This would, in turn, permit informed extensions/enhancements to the model/analysis by virtue of possessing knowledge of how they were developed from the outset. For example, it is not uncommon in the modelling community to pick up published models from any of the various model repositories, reuse these to arrive at further inferences and conclusions, without really being aware of the model’s origin, scope or limitations. This is very often attributed to the difficulty in locating or accessing the data underlying model development.

Another common problem is with regards to difficulty in reproducing results reported in publications. This can be simply owing to unavailability of the model source code or lack of simulation/protocol specific details being provided in the publications, that are needed to allow reproducibility. An extreme case of this problem was posed by *ReScience C*, a journal that encourages testing the reproducibility/replicability of computational methods based solely on the corresponding published article. In 2020, they launched a “Ten Years Reproducibility Challenge” where scientists were asked to reproduce their own computational work published at least 10 years earlier (Perkel 2020). The purpose of this challenge was to highlight the difficulties involved in even reproducing one’s own work (let alone that of others), the need for reliable storage of all relevant modelling resources, and proper documentation.

It is common practice in current times for journal publishers to demand a statement on data availability from the authors prior to publication (Hofer et al. 2019; Hrynaszkiewicz 2019). This certainly does encourage authors to make underlying data resources available to the scientific community. But what is lacking is a structured and systematic way of offering these resources. Very often authors simply resort to stating something similar to “*Data are available from the authors upon request*”. Studies have reported that it is uncommon for published articles to contain the underlying resources, or offer links to access them (Nüst et al. 2018; Stagge et al. 2019). As may be imagined, this often results in a situation where users interested in a particular computational work face a brick wall because authors are unresponsive, have left academia, or because code or data have been lost or are otherwise no longer available.

With the concept of Live Papers presented here, we intend to establish a platform by means of which authors can easily aggregate the various data components underlying their computational study into a systematic, structured and distributable format. The live paper builder tool has been developed with the primary focus on making the data sharing process as simple as possible. The curation process ensures that resources are hosted on reliable data storage services, either by transferring the specified resources to the EBRAINS archival data repository, or by linking to other established neuroscience repositories. This helps tackle the issue of long term retrievability of publication related resources. Other additional resources, such as Jupyter notebooks, allow for enhanced documentation of simulation protocols or data analysis pipelines by demonstrating how various simulations were undertaken. With data being managed via the EBRAINS KG, the issue of data provenance can also be better tackled, along with tighter integration with other tools and services offered under the EBRAINS ecosystem.

One apparent limitation of the live papers is the lack of standardisation of the content of the live papers. In its current form, the authors are free to determine what resources they wish to provide in the manuscript. This is in part to encourage wider uptake by not imposing rigid requirements, but also borne out of the need for handling the diversity in computational studies, where the set of employed resources can greatly vary. Currently, each live paper submission is verified to ensure that all input resources are actually accessible, functional, and hosted on a reliable data storage repository. Live papers can be password-protected, so they can be shared with reviewers of an associated manuscript prior to publication. Reviewers would be best placed to identify and recommend what missing features should be made available in the live paper. We have already had instances where live papers have been used to furnish resources and other details demanded during the review phase.

### Comparison with Other Reproducibility Efforts

(Konkol et al. 2020) undertook a review of several applications and services created with the purpose of furthering transparent and reproducible research. These included applications such as Authorea, Binder, eLife Reproducible Document Stack (RDS) - extended recently to a web-native format with eLife Executable Research Articles (ERAs), and ReproZip (in combination with ReproServer), amongst many others. A large number of stakeholders are involved in the scientific process - publishers, editors, authors, readers, reviewers, and librarians. Each group comes with their own set of requirements and considerations, and the authors therefore found it infeasible and inappropriate to provide a ranking for these reviewed applications as each satisfies user-specific requirements to varying extents.

(Konkol et al. 2020) reported that, though many of these applications were in active usage at the time of reporting, it might take greater effort to have these accepted and adopted into publishers’ infrastructures. Also, journals and publishers often differ in the formats of accepted submissions, and this transformation is often non-trivial. The authors therefore suggest that it might be simpler to have reproducible documents as a supplementary resource to the actual publication, especially for the immediate future, before a transition is successfully made by both researchers and publishers to have manuscript embedded reproducible elements. The concept of live papers, presented here, provides exactly such a supporting document associated with a published article.

Most of the applications in their study were found to make use of literate programming to support reproducible research. (Konkol et al. 2020) importantly point out that the range of programming expertise varies widely between user groups—“from trained research software engineers to self-taught beginners”. The learning curve involved in adopting such applications in the publication workflow can often be a huge deterrent to their uptake. Some of the applications are commercial and therefore require authors to have paid accounts to access all features. Also, some applications restricted users to open licenses for the shared content and some of them didn’t have an online version and required users to host it themselves, thereby adding to the technical overhead. Moreover, regardless of whether the considered applications can be self-hosted or not, they require installation, configuration and maintenance operations in addition to quota management and resource monitoring. Finally, those applications usually require a previous registration to the hosting platform for accessing the resources. In a review process, this could prevent a double-blind procedure in that the reviewers would not be able to visualize the material anonymously. In concluding, the authors of the study also strongly urged research authors to publish material resources in well-established, reliable repositories that guarantee long-term availability of these resources, in addition to an executable version using any of the reproducibility supporting applications.

The primary objective underlying the concept of live papers is twofold: 1) to offer a human-friendly and content-rich platform to the scientific community for accessing resources related to neuroscientific publications; 2) to provide authors, aiming at (or required to) share data, models and methods adopted in their manuscripts, with a flexible and easy-to-use environment able to reduce the overhead of the publishing-and-sharing process —in terms of time, effort, and cost. All of these are well-known obstacles that authors need to face in the path of open and reproducible research. This in part justifies the choice of a low entry threshold for developing live papers, whereby the authors decide what resources are made available, and the curation process for publication, for the moment, simply ensures that these resources are accessible, functional and made available long-term.

A key and novel feature of the Live Papers is its capacity to complement existing data storage repositories by working as an aggregator of resources from established platforms (by linking them together), while offering both its own data storage capabilities and dedicated tools and services for the exploration and use of the resources made available. For example, a neural data visualiser is seamlessly integrated in the Live Papers for reading and displaying all the neural data type supported by the Neo library and only requires the authors to specify the url of the data source. The same holds true for 3D visualization of neural morphologies and model data and metadata access. In addition to the user-friendliness of the Live Papers webpage, the insertion of online resources is made even easier thanks to an ad hoc developed search engine that is able to query and fetch content data, based on keywords inserted by the users, from several online scientific repositories (e.g., ModelDB, OSB, EBRAINS KG). Differently from other applications, the Live Papers web interface is extremely lightweight, in that it consists in an easy-to-maintain JavaScript-based web frontend; hence, no specific installation and configuration are required to authors, publishers and developers. At the same time, being part of the EBRAINS Research Infrastructure, the Live Papers take advantage of the rich ensemble of tools and services it offers: 1) Jupyter notebooks can be created and configured in the EBRAINS Collaboratory environment and linked to the Live Papers; 2) the Live Papers platform is integrated with the BlueNaas simulator engine (https://ebrains-cls-interactive.github.io/online-use-cases.html#/single_cell_insilico_experiments) via a web-socket communication channel; with a little programming effort a NEURON model can be run without any specific installation and the results shown in the user’s browser; 3) if needed and duly justified, authors can request dedicated HPC resources available in the EBRAINS framework for running demanding operations/simulations; 4) long-term data repositories are offered to the Live Papers creators. Thanks to this tight integration, Live Papers delegate the burden of software development and maintenance to external services keeping the interface easily accessible and maintainable.

In addition, Live paper documents are built separate from the article manuscript, thereby allowing authors to follow the traditional approach to manuscript preparation, while being able to work in parallel on the supplementary live paper document. Once the resources are uploaded online, a live paper of moderate complexity, such as most of those currently available on the platform, can be developed in a matter of a few hours and does not require any specific programming skill. Also, in a double-blind review scenario, the document can be password-protected thus guaranteeing an exclusive access without any requirement for user registration. Finally, the authors of live papers are free to specify the licensing policy for the resources that they wish to share.

It should also be noted that the issue of reproducibility is a much greater challenge requiring technical interventions that tackle differences in hardware, operating systems, versioning, and so on. As briefly stated above, many of the available applications, along with tools such Sumatra, CDE, and NoWorkflow, attempt to address these matters through different approaches. With the concept of Live Papers presented here, the issue being addressed is tied more closely to data availability, which can be considered as a prerequisite, or a first step, for any kind of reproducibility effort.

### Future Developments

We intend to implement more features within the live papers based on community feedback and requirements. One such feature that we are currently working towards is the ability to launch NEURON (Hines and Carnevale 1997) based models using cloud services, through which NEURON parameters and files can be configured and utilized, from within the live papers. Provisions for enabling such advanced or custom functionalities already exist and some published live papers already leverage a web-socket based service to remotely run NEURON models using the BlueNaaS application; nonetheless this requires web development skills. In future we hope to offer a user-friendly interface and new widgets for this functionality and extend it to other simulators as well, once the development of corresponding cloud-based services will be mature enough. This would eliminate the need to download models and run them in a suitable simulation environment. We also plan to extend the data analysis capability of the Live Paper documents. For example, while the neural data visualiser is transparently integrated in a live paper by simply linking the data source, no functionality is currently offered for the analysis of the displayed electrophysiological recordings. We plan to add a dedicated widget/panel that allows to extract the most significant measures from the neural data (e.g., mean firing rate, number of spikes, inter-spike interval values) and, eventually, further extend this functionality to more specific analysis, depending on the community requests. In case a simulation panel is integrated in the live papers, as envisioned above, such a tool would also be instrumental for the analysis of the simulated activity.

The Live Paper platform was initially setup in 2018, primarily targeting publications arising from the Human Brain Project. The platform currently hosts over twenty live papers associated with scientific publications. This initial release enabled us to identify shortcomings and incorporate features that were found to be essential for a better user experience in terms of both utility and ease of use. Having been successfully tested, we now intend to roll this out to the wider scientific community, and hope to see widespread adoption. The concept of live papers presented here is readily applicable to scientific studies more broadly, and need not be restricted to neuroscience, although the current implementation is mostly oriented to the neuroscientific field. At its core, the live papers are simply a means for effectively disseminating scientific resources, to help further research in a collaborative environment. Most scientific disciplines can benefit from such a service, and therefore the concept of live papers holds immense promise and potential.

In summary, Live Papers are intended to be a structured and interactive supplementary document, either to complement a journal publication or as stand-alone resource, that allows users to readily access, explore and reuse the various kinds of code and data underlying scientific studies.

## Data Availability

The EBRAINS Live Papers platform is entirely open-source, with the code being available on GitHub (https://github.com/appukuttan-shailesh/ebrains-live-papers). This includes both the live paper viewer and the live paper builder tools. All live paper resources are publicly accessible, via the live paper platform, without requiring any registration or authentication. The use of resources hosted in each live paper is governed by the license specified by the authors of that specific live paper.
